# Effects of Garlic Oil and Cinnamaldehyde on Sheep Rumen Fermentation and Microbial Populations in Rusitec Fermenters in Two Different Sampling Periods

**DOI:** 10.3390/ani14071067

**Published:** 2024-03-30

**Authors:** Jairo García-Rodríguez, Cristina Saro, Iván Mateos, María Dolores Carro, María José Ranilla

**Affiliations:** 1 Departamento de Producción Animal, Universidad de León, Campus de Vegazana, s/n, 24071 León, Spain; jgarr@unileon.es (J.G.-R.); csarh@unileon.es (C.S.); imata@unileon.es (I.M.); 2 Instituto de Ganadería de Montaña, CSIC—Universidad de León, Finca Marzanas, s/n, 24346 Grulleros, Spain; 3 Departamento de Producción Agraria, Escuela Técnica Superior de Ingeniería Agronómica, Agroalimentaria y de Biosistemas, Universidad Politécnica de Madrid, Ciudad Universitaria, 28040 Madrid, Spain; mariadolores.carro@upm.es

**Keywords:** garlic oil, cinnamaldehyde, Rusitec, methane, qPCR, ARISA, microbial protein synthesis, rumen fermentation, sheep

## Abstract

**Simple Summary:**

Garlic oil and cinnamaldehyde are plant-derived bioactive compounds with antimicrobial activity, which might contribute to improving the efficiency of rumen fermentation. However, an apparent adaptation of microbial populations to these compounds is usually observed over time. In this study, the effects of garlic oil and cinnamaldehyde on rumen fermentation and microbial populations were assessed in two periods of time using an in vitro system (Rusitec fermenters). Despite this possible adaptation, garlic oil supplementation reduced methane production and enhanced the energy profile of fermentation end products over time, whereas cinnamaldehyde appeared to increase the microbial protein supply to the animal. Thus, the use of these compounds as feed additives could contribute to increasing the efficiency and sustainability of ruminant production systems, bringing both economic and environmental benefits.

**Abstract:**

Garlic oil (GO) and cinnamaldehyde (CIN) have shown potential to modify rumen fermentation. The aim of this study was to assess the effects of GO and CIN on rumen fermentation, microbial protein synthesis (MPS), and microbial populations in Rusitec fermenters fed a mixed diet (50:50 forage/concentrate), as well as whether these effects were maintained over time. Six fermenters were used in two 15-day incubation runs. Within each run, two fermenters received no additive, 180 mg/L of GO, or 180 mg/L of CIN. Rumen fermentation parameters were assessed in two periods (P1 and P2), and microbial populations were studied after each of these periods. Garlic oil reduced the acetate/propionate ratio and methane production (*p* < 0.001) in P1 and P2 and decreased protozoal DNA concentration and the relative abundance of fungi and archaea after P1 (*p* < 0.05). Cinnamaldehyde increased bacterial diversity (*p* < 0.01) and modified the structure of bacterial communities after P1, decreased bacterial DNA concentration after P2 (*p* < 0.05), and increased MPS (*p* < 0.001). The results of this study indicate that 180 mg/L of GO and CIN promoted a more efficient rumen fermentation and increased the protein supply to the animal, respectively, although an apparent adaptive response of microbial populations to GO was observed.

## 1. Introduction

Worldwide demand for livestock products is expected to increase substantially in the coming years, with ruminants being a key element in sustainable agriculture [1]. These animals have the ability to transform useless food constituents for humans, such as fiber, into edible food as a result of the activity of the complex rumen microbial ecosystem, which is composed of bacteria, protozoa, fungi, and archaea [2]. Rumen microbial populations degrade and metabolize feed to produce volatile fatty acids (VFAs) and microbial protein, which are important sources of energy and protein for ruminants [3]. However, rumen fermentation has energy and protein inefficiencies due to methane production and ammonia-N losses (NH_3_-N), respectively, which can negatively affect animal performance and contribute to increased environmental pollution [3]. Thus, optimizing rumen fermentation to enhance animal production is an important goal in ruminant nutrition, which could contribute to reducing the environmental impact of ruminant production systems at the same time.

Bioactive compounds derived from plants are secondary metabolites that contain chemical constituents and functional groups with antimicrobial activity [3], and they have been studied due to their potential as alternatives to growth-promoting antibiotics in ruminants [1]. Among bioactive plant compounds, essential oils are volatile and aromatic lipophilic components that contain functional groups such as terpenoids, phenolics, organosulfur compounds, or homologues of phenylpropanoids [1]. Garlic oil (GO) and cinnamaldehyde (CIN) are two of the most studied essential oils as rumen fermentation modifiers.

Garlic oil is a complex mixture of organosulfur compounds, including alliin, allicin, diallyl sulfide, diallyl disulfide, and diallyl trisulfide [4,5]. Antimicrobial activity of compounds from GO have shown antibacterial, antifungal, and antiprotozoal effects [1,6,7], and therefore, the effects of GO and their compounds have been assessed as potential rumen microbiome modifiers [1]. The supplementation of continuous fermenters fed a diet for dairy animals did not affect nutrient disappearance and VFA production, whereas methane production and the acetate/propionate ratio decreased [8,9,10], as well as in 24 h fermentations in batch cultures [9,11,12]. Moreover, 500 mg of GO/L reduced archaea abundance in batch cultures [13,14] without affecting that of bacteria, although the effects on the abundance of the main rumen fibrolytic bacteria (*Fibrobacter succinogenes*, *Ruminococcus flavefaciens*, and *Ruminococcus albus*) and protozoa showed controversial results among studies [13,14,15,16].

Cinnamaldehyde is the main component of cinnamon essential oil, and it is a phenylpropanoid with antimicrobial activity against bacteria [3,17]. Although studies focused on the effects of CIN on rumen fermentation have shown more variable results than those focused on GO, CIN appears to mainly affect nitrogen metabolism in the rumen [11,17,18,19]. Cinnamaldehyde reduced acetate proportion in batch and continuous cultures [10,11,18,20] as well as NH_3_-N production [11,18] without any effects on nutrients digestibility and total VFA production. Moreover, Cantet et al. [19] observed a greater conversion efficiency of dietary N into milk protein N after supplementing the diet of dairy cows with 125 mg of CIN/d. Despite the effects of both GO and CIN on rumen fermentation, a possible adaptation of rumen microbial populations to garlic, cinnamon, or oregano essential oils has been described [1,21], causing a reduction in their effectiveness.

To the best of our knowledge, no study has evaluated the effects of GO and CIN on rumen fermentation and microbial populations in two different periods of time in fermenters or in vivo. Moreover, although previous in vitro studies [6,9,10] have assessed the effects of these compounds on rumen fermentation, information on their influence on microbial protein synthesis (MPS), enzymatic activity, and microbial populations is less available. Furthermore, previous results have been highly variable among studies due to differences in diets, type of study (in vitro vs. in vivo), supplementation time, or the type, composition, and dose of the essential oils used in the different studies, among others. We hypothesized that GO and CIN have potential to positively modify in vitro rumen fermentation and microbial populations, but their effectiveness is less marked over time. Thus, the aim of this study was to assess the effects of adding 180 mg/L of GO and CIN on rumen fermentation parameters, enzymatic activity, MPS, and microbial populations in Rusitec fermenters fed a mixed diet for dairy sheep (50:50 forage/concentrate), as well as whether these effects were observed in two different periods of time.

## 2. Materials and Methods

### 2.1. Donor Animals and Diet

Ruminal contents (solid and liquid) of four rumen-cannulated non-lactating Merino sheep (56.3 ± 1.56 kg of body weight (BW)) were used to inoculate the Rusitec fermenters. Sheep care and rumen content withdrawal were done by skilled staff according to the Spanish guidelines for experimental animal protection (Royal Decree 53/2013 of February 1st on the protection of animals used for experimentation or other scientific purposes). Donor sheep were fed a mixed diet composed of 500 g of alfalfa hay and 500 g of concentrate per kg DM, distributed at a fixed rate of 42 g of dry matter (DM) per kg of BW^0.75^ in two equal meals at 9:00 and 18:00 h. The same diet was incubated in the Rusitec fermenters, and its ingredients and chemical composition are shown in Table 1.

### 2.2. Additives

The additives used in the present study were commercial products supplied by Axiss France SAS (Bellegarde Sur Valserine, France). The GO was composed of 0.65 g diallyl disulfide, 0.15 g diallyl trisulfide, and 0.10 g allicin per g of oil, and CIN had 99% purity. Both additives were added into the fermenters at a final concentration of 180 mg/L from the second day until the end of the trial. This concentration was selected based on the results obtained in a batch culture experiment conducted with the same additives and a similar diet [11]. The daily amount of each additive was dissolved in 0.7 mL of ethanol and was supplied each day directly into the liquid of the fermenters at feeding time. Control (CON) fermenters received 0.7 mL of ethanol daily.

### 2.3. Experimental Procedures

Two 15-day identical incubation runs were carried out using six Rusitec fermenters (600 mL of effective volume). In each run, treatments were assigned randomly, with two fermenters receiving each of the three experimental treatments (180 mg/L of GO, 180 mg/L of CIN, or CON). The general incubation procedure was carried out, as described by Martinez et al. [22], and additive supplementation started on the second day of incubation. All fermenters received 15 g DM of forage and 15 g DM of concentrate daily, which were supplied in separate nylon bags (100 μm pore; 8 × 15 cm). On the first day of each incubation run, ruminal contents of each sheep were collected immediately before the morning feeding and were mixed and strained through four layers of cheesecloth. Solids and liquids were collected separately in pre-warmed thermal flasks that were transported to the laboratory. The pH of rumen fluid was measured, and each fermenter was filled up with 200 mL of ruminal fluid and 250 mL of artificial saliva (pH = 8.4) [23]. Then, one bag with forage, one bag with concentrate, and one bag containing 80 g of solid rumen content were introduced into each fermenter. Bags containing solid rumen content and undigested concentrate were removed after 24 h and were replaced by two new bags containing forage and concentrate, respectively. On each of the following days, the nylon bags with the undigested residues of concentrate and forage were taken out after 24 h and 48 h of incubation, respectively, and replaced by two new bags. Fermenters were manipulated daily under atmospheric conditions, and they were closed and flushed with N_2_ after manipulation. The daily infusion rate of artificial saliva was set at 650 mL (dilution rate of 4.51% per h) to resemble values observed in sheep in previous studies [24]. Saliva was infused using a peristaltic pump, and effluents were collected in bottles that contained 20 mL of H_2_SO_4_ at 20% *v*/*v*.

**Figure 1:**
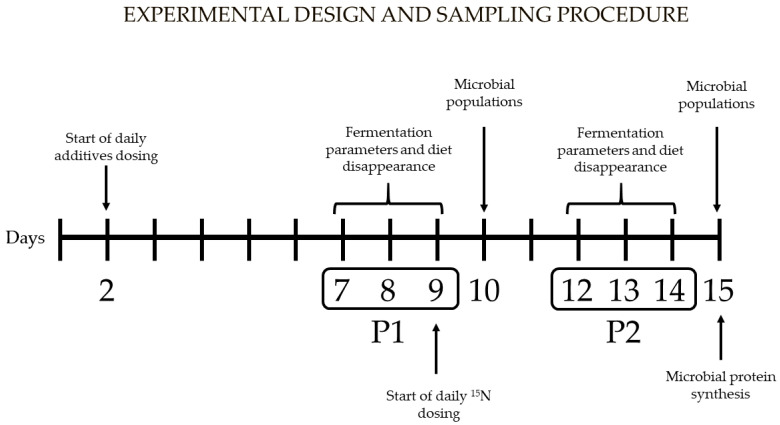
Experimental design and sampling procedure carried out in each incubation run. Samples to assess rumen fermentation parameters, diet disappearance, and enzymatic activity were taken in P1 (7 to 9 days of incubation) and P2 (12 to 14 days of incubation), samples to study microbial populations were collected the day after P1 (day 10) and P2 (day 15), and those to determine microbial protein synthesis were taken on the last day of incubation (day 15).

Figure 1 represents the experimental design and the sampling procedure carried out in each incubation run. Within each run, samples to assess fermentation parameters and diet disappearance were taken in two sampling periods, namely P1 (7 to 9 days of incubation) and P2 (12 to 14 days of incubation), whereas samples to study microbial populations were collected the day after each of these periods (days 10 and 15). Furthermore, the samples used to determine MPS were taken on the last day of incubation (day 15).

In each day of P1 and P2, the gas produced from each fermenter was quantified (model TG1; Ritter Apparatebau GmbH, Bochum, Germany), and a sample (10 mL) was taken into vacuum tubes for methane analysis. On the same days, about 5 mL of effluent were collected and frozen (−20 °C) for analysis of volatile fatty acid (VFA) and ammonia-N (NH_3_-N) concentrations, and 5 mL of liquid content from each fermenter were placed into cryovials, which were immediately frozen (−80 °C) until the analysis of amylase, xylanase, and endoglucanase activities. Finally, the nylon bags taken out from the fermenters during these days were washed (cold rinse cycle of a washing machine; 20 min), dried (60 °C; 48 h), and weighed to determine the diet’s apparent disappearance, as described by Martínez et al. [22], which was calculated as the sum of disappearance of both forage and concentrate using the bags collected from the same fermenter on the same incubation day. Additionally, the following day after both P1 and P2, samples from the liquid content of the fermenters (LIQ) and solid residues of the nylon bags (solid digesta; SOL) were collected into sterile cryovials, which were immediately frozen at −80 °C for the study of bacterial diversity and microbial populations.

A solution of ^15^NH_4_Cl (Sigma-Aldrich Quimica S.L., Madrid, Spain) was added to the artificial saliva (4.0 mg of ^15^N/g of dietary N) during the last six days of each incubation run in order to label the bacteria for measuring MPS. On the last day of incubation, both the effluent and the solid content of the nylon bags withdrawn from the fermenters were taken for measuring MPS, as described by Carro and Miller [25]. About 500 mL of effluent were used to isolate pellets of liquid-associated bacteria by differential centrifugation [26], and the rest of the effluent was freeze-dried for quantifying DM content and ^15^N enrichment. The solid contents of the two nylon bags taken from each fermenter were carefully mixed. A subsample (about 0.2 of total sample) was used for the determination of DM content and ^15^N, and another subsample was placed into cryovials for analysis of microbial populations, as described before. The rest of the solid content was treated with a saline solution of 0.1% methylcellulose before the isolation of solid-associated bacteria. as detailed by Ramos et al. [26].

### 2.4. Analysis of Bacterial Diversity and Characterization of Microbial Populations

DNA extraction was performed in triplicate from the pellets obtained after centrifugation of 1 mL of LIQ (20,000× *g*, 5 min, 4 °C) and from 200 mg of DM of lyophilized SOL samples using the procedure of Yu and Morrison [27]. The concentration and purity of DNA were measured in a Nanodrop ND-1000 (NanoDrop Technologies, Wilmington, DE, USA). The absorbance ratios (A260:A280) were between 1.83 and 1.96.

For Automated Ribosomal Intergenic Spacer Analysis (ARISA), the universal bacterial primers 16S-1392F and 23S-125R [28] were used for amplifying the internal transcribed spacer of DNA, according to Saro et al. [29], using a 2720 Thermal Cycler (Applied Biosystems, Foster City, CA, USA) for thermocycling. An ABI Prism 3130 Genetic Analyzer (Applied Biosystems, Foster City, CA, USA) was utilized for the automated detection of ARISA fragments. Software GeneMarker v1.80 was used for the identification of peaks, which was performed using an internal size standard. The presence or absence of peaks was utilized to create a dissimilarity matrix for the comparison of electropherograms profiles. A principal coordinate analysis (PCoA) based on the Bray–Curtis distances was performed to analyze potential differences among samples using the R package vegan [30]. The diversity of bacterial communities was assessed using Shannon’s diversity index [31].

Total bacterial and protozoal DNA concentrations, as well as the relative abundance of *Fibrobacter succinogenes*, *Ruminococcus flavefaciens*, *Ruminococus albus*, fungi, and methanogenic archaea in relation to total bacteria, were determined using quantitative PCR (qPCR). The primers used for bacteria, fungi, *F. succinogenes*, and *R. flavefaciens* were those described by Denman and McSweeney [32]. The primers used to determine protozoa and archaea were defined by Sylvester et al. [33] and Denman et al. [34], respectively, whereas the primer used to quantify *R. albus* was described by Koike and Kobayashi [35]. Each qPCR reaction mixture contained 10 µL of SYBR Green PCR Master Mix (Applied Biosystems, Warrington, UK), 0.9 µL of each primer (20 µM), 6.2 µL of milli-Q water, and 2 µL of extracted DNA. The cycling conditions were 94 °C for 10 min for denaturation and 40 cycles of 95 °C for 15 s and 60 °C for 1 min for bacteria, *R. albus*, *R. Flavefaciens*, *F. succinogenes*, fungi, and archaea. For protozoa, the cycling conditions were 94° C for 10 min for denaturation and 40 cycles of 95 °C for 15 s, 54 °C for 30 s, and 60 °C for 1 min. To determine the specificity of amplification, analysis of product melting was performed after each amplification, increasing the temperature at a rate of 0.3 °C every 30 s from 60 to 95 °C. Bacterial and protozoal DNA extracted from microbial pellets collected from the rumen content of sheep, according to Saro et al. [36], were used as a standard for absolute quantification of bacteria and protozoa. All qPCR reactions were performed in duplicate using an ABI PRISM 7000 Sequence Detection System (Applied Biosystems, Warrington, UK), as described by Saro et al. [36], and PCR efficiencies ranged from 92.2% to 107.5%.

### 2.5. Analytical Procedures

Dry matter (DM; ID 934.01), ash (ID 930.05), nitrogen (N; ID 978.04), and ether extract (ID 930.09) contents were determined according to the Association of Official Analytical Chemists [37]. Analysis of neutral detergent fiber (NDF) and acid detergent fiber (ADF) was performed following the procedure of Van Soest et al. [38], using an ANKOM^220^ Fiber Analyzer unit (ANKOM Technology Corporation, Fairport, NY, USA), and samples were ground through a 1 mm screen. The NDF analyses were conducted using sodium sulphite and heat-stable amylase, and both fiber fractions were expressed excluding residual ash.

The concentrations of VFA and NH_3_-N in the effluents were analyzed, as described by Martinez et al. [39], and methane concentrations were assessed by gas chromatography (Shimadzu GC 14B; Shimadzu Europa GmbH, Duisburg, Germany), as proposed by Martinez et al. [40]. Amylase, xylanase, and endoglucanase activities in the fermenters were assessed following the colorimetric procedures described by Giraldo et al. [41], using soluble starch, oat spelt xylan, and carboxymethylcellulose as substrates, respectively.

### 2.6. Calculations and Statistical Analyses

Calculations of MPS using ^15^N as an external microbial marker have been detailed by Carro and Miller [25]. The organic matter (OM) apparently fermented was estimated from daily VFA production, as proposed by Demeyer [42], and was used to calculate the efficiency of MPS. The relative abundance of *F. succinogenes*, *R. flavefaciens*, *R. albus*, fungi, and methanogenic archaea DNA was determined relative to the absolute quantification of total bacteria as 2^−(CT target − CT total bacteria)^, where C_T_ represents the threshold cycle after correcting for differences in amplification efficiencies between the target and total bacteria. Correction factors for the relative qPCR efficiency of *F. succinogenes*, *R. flavefaciens*, *R. albus*, fungi, and archaea were 1.030, 0.977, 0.997, 1.005, and 1.059, respectively.

All statistical analyses were done using the PROC MIXED of the SAS package (SAS Institute Inc., Cary, NC, USA). A mixed model with repeated measures was used to analyze data on fermentation parameters, enzymatic activity, and diet disappearance, including the treatment, incubation run, time, and treatment x time interaction as fixed effects and the fermenter as a random effect. The analysis of data on MPS, bacterial diversity, and microbial abundance was performed as a mixed model, including the treatment and incubation run as fixed effects and the fermenter as a random effect. Contrasts were performed under the following principle: When a significant effect of additive was detected within period (P1 and P2), each additive was compared with the control by Dunnett test. Significant differences were considered at *p* < 0.05.

## 3. Results

### 3.1. Diet Disappearance, Rumen Fermentation Parameters, and Enzymatic Activity

Table 2 and Table 3 show the effects of the experimental treatments on diet disappearance, fermentation parameters, and enzymatic activities in P1 and P2, respectively. Garlic oil decreased ADF disappearance in P1 (*p* = 0.01) but not in P2, without affecting DM, OM, or NDF disappearance in any sampling period (*p* > 0.05). Garlic oil supplementation did not affect pH, total VFA, and NH_3_-N daily production in the fermenters, neither in P1 nor in P2 (*p* > 0.05). In contrast, GO decreased the proportions of acetate, butyrate, isovalerate (*p* < 0.01), and the acetate/propionate ratio (*p* < 0.001) and increased the proportions of propionate, valerate, and caproate (*p* < 0.001) in both sampling periods. Isobutyrate proportion was unaffected in P1 (*p* > 0.05) and increased in P2 (*p* < 0.01) after GO addition. Moreover, GO reduced methane production (*p* < 0.001) and the methane/VFA ratio in both sampling periods (*p* = 0.02). No effects of GO were observed on the enzymatic activities of amylase, xylanase, and endoglucanase in any sampling period (*p* > 0.05).

Cinnamaldehyde supplementation did not affect DM, OM, and FND disappearances in any sampling period (*p* > 0.05), although it increased ADF disappearance in P2 (*p* = 0.02), compared to CON fermenters. Total VFA and NH_3_-N daily productions and pH were unaffected (*p* > 0.05) by CIN in any sampling period. The addition of CIN did not affect individual VFA proportions in P1 (*p* > 0.05). However, a lower propionate molar proportion (*p* < 0.001), greater isovalerate proportion, and an increased acetate/propionate ratio (*p* < 0.01) were observed in P2 after CIN addition. Moreover, CIN did not affect methane production in either P1 or P2 (*p* > 0.05). In contrast, the addition of CIN in the fermenters increased the methane/VFA ratio (*p* = 0.03) in P1, although this effect was not observed in P2 (*p* > 0.05). Cinnamaldehyde supplementation did not affect amylase, xylanase, and endoglucanase enzymatic activities in the fermenters in any sampling period (*p* > 0.05).

### 3.2. Microbial Protein Synthesis (MPS), Bacterial Diversity, and Microbial Populations

As shown in Table 4, MPS and its efficiency were unaffected (*p* > 0.05) by GO supplementation. Conversely, CIN increased MPS in the solid digesta (*p* = 0.01) but not in the effluent (*p* > 0.05). Thus, fermenters that were supplemented with CIN showed greater total MPS (*p* < 0.001) and MPS efficiency (*p* < 0.01) than CON ones.

Results of bacterial diversity and microbial populations in SOL and LIQ phases of the fermenters at the end of P1 and P2 are shown in Table 5 and Table 6, respectively. No effects of GO were observed on the number of peaks, Shannon’s diversity index, and the concentration of bacterial DNA, either in the SOL phase or in the LIQ phase, on any sampling day (*p* > 0.05). Compared with CON fermenters, GO reduced protozoal DNA concentration in both digesta phases at the end of P1 (*p* < 0.05), as well as the relative abundance of fungi (*p* = 0.01) and archaea (*p* = 0.02) in SOL and LIQ, respectively, without any effect on that of *F. succinogenes*, *R. flavefaciens*, and *R. albus* (*p* > 0.05). In contrast, GO supplementation reduced the relative abundance of *F. succinogenes* in the SOL phase at the end of P2 (*p* < 0.01), whereas the abundance of the rest of the microbial groups and bacterial species analyzed was unaffected by the additive on the same sampling day (*p* > 0.05).

The addition of CIN did not affect bacterial diversity in the SOL phase in any sampling day (*p* > 0.05). However, the number of peaks and Shannon’s diversity index in the LIQ phase increased at the end of P1 after CIN addition (*p* < 0.01), although these effects were not observed at the end of P2 (*p* > 0.05). Supplementation with CIN reduced protozoal DNA concentration in the SOL phase at the end of P1 (*p* < 0.01), without affecting that of bacteria in any phase on the same sampling day (*p* > 0.05). In contrast, bacterial DNA concentration was reduced by CIN addition in the SOL phase at the end of P2 (*p* = 0.03), as well as that of protozoa in the LIQ phase (*p* < 0.05). The relative abundance of fungi, archaea, *F. succinogenes*, *R. flavefaciens*, and *R. albus* was unaffected by CIN, either in the SOL phase or in the LIQ phase, on any sampling day (*p* < 0.05).

**Figure 2:**
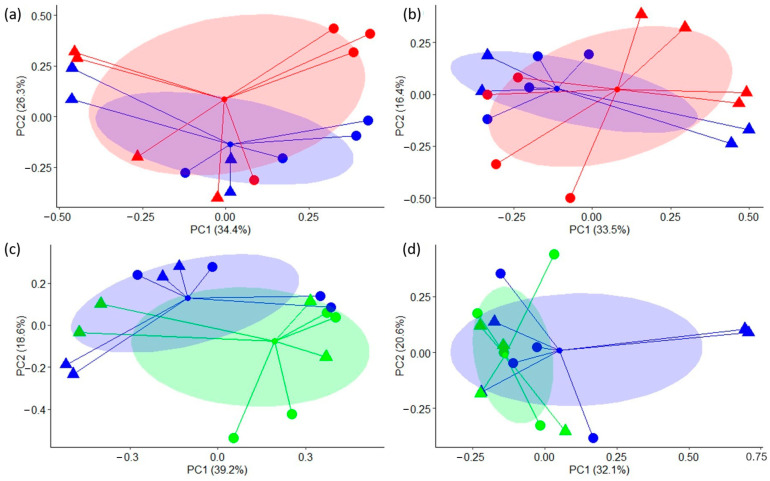
Principal coordinate analysis (PCoA) based on Bray–Curtis distance matrix of the automated ribosomal intergenic spacer analysis (ARISA) profiles from liquid (circles) and solid (triangles) phases of control fermenters (blue) or those supplemented with garlic oil (red) or cinnamaldehyde (green). (**a**) Control vs. garlic oil at the end of P1 (day 10); (**b**) control vs. garlic oil at the end of P2 (day 15); (**c**) control vs. cinnamaldehyde at the end of P1 (day 10); (**d**) control vs. cinnamaldehyde at the end of P2 (day 15).

Figure 2 represents the PCoA plots based on Bray–Curtis distances and consists of four different plots, namely a, b, c, and d. Plots 2a (at the end of P1) and 2b (at the end of P2) compared GO (red) and CON (blue) samples, with the percentage of variance explained by coordinates 1 and 2 being 34.4% and 26.3% for plot 2a and 33.5% and 16.4% for plot 2b, respectively. These plots indicate that most of samples were separated according to digesta phase by coordinate 1 on both sampling days, whereas no clear separation was observed between samples from CON and GO-fermenters. The plots 2c and 2d compared CIN (green) and CON (blue) samples at the end of P1 and P2, respectively, with the percentage of variance explained by coordinates 1 and 2 being 39.2% and 16.8% for plot 2c and 32.1% and 20.6% for plot 2d, respectively. Samples appear to be separated not only according to digesta phase but also according to treatment by coordinate 1 after CIN supplementation at the end of P1, although no clear separation of samples was observed at the end of P2.

## 4. Discussion

### 4.1. Garlic Oil

Garlic oil addition decreased ADF disappearance in P1 in this study, which could be related to the decrease in the amount of protozoal DNA caused by this additive at the end of P1, as well as in the relative abundances of fungi and archaea in SOL and LIQ phases, respectively. It should be noted that the effects of GO on nutrient disappearance and microbial populations in P1 were not observed in P2, suggesting a possible adaptation of microorganisms to the additive. Despite this potential adaptation, in vitro rumen fermentation pattern changed after adding 180 mg/L of GO in Rusitec fermenters, mainly by reducing methane production and acetate/propionate and methane/total VFA ratios in both sampling periods. The reduction in methane production caused by GO is consistent with the decrease in acetate/propionate ratio, since hydrogen is released during acetate production and might be used by archaea for methanogenesis. As propionate is known to be the major precursor of glucose in ruminant metabolism, the changes caused by GO addition in the molar proportions of individual VFA could be considered energetically more favorable to the animal. Additionally, lower methane/VFA ratios have also been previously observed in batch cultures after GO addition [9], suggesting that this additive might increase the energy obtained by the host animal per unit of fermented substrate. Thus, the decreased acetate/propionate and methane/total VFA ratios after GO addition observed in both sampling periods P1 and P2 would result in a more efficient fermentation, which can be considered positive changes in the rumen fermentation pattern. Furthermore, enzymatic activities, MPS, bacterial DNA concentration, and bacterial diversity were not affected by GO in the present study, suggesting that this compound could positively modify in vitro rumen fermentation patterns without negative effects on nutrient disappearance, microbial growth, and bacterial populations.

In agreement with the results of this study, previous in vitro studies reported that GO addition did not affect nutrient disappearance and total VFA production and reduced methane production and acetate/propionate ratios in continuous cultures fed a diet for dairy animals (50:50 forage/concentrate) and supplemented with 300 mg GO/L for 6 to 10 days [8,9,10]. Similar results were observed in 24 h fermentations in batch cultures after adding 180 mg of GO/L [11].

Concerning enzymatic activities, lower doses of GO (83.3 and 166.7 µL/L; [43]) and garlic extract (8.3 and 16.7 mL/L; [44]) were reported to decrease xylanase and carboxymethylcellulase activities in 24 h incubations in batch cultures, with these effects attributed to the antimicrobial effect of garlic compounds. These results contrast with the results of this study. Nonetheless, differences in the composition of the GO or garlic extracts and in the in vitro systems used in the different studies can help explain these contrasting results, since the fermenters have an inlet and outlet flow that could modify GO concentrations as they occur in the rumen, whereas there is no outflow in batch cultures.

Both MPS and its efficiency were unaffected by GO in this study, which agrees well with previous studies on continuous cultures using 300 mg/L of GO and purine bases as microbial markers [9,10]. This is a positive result, since microbial protein is an essential source of amino acids for ruminants. The absence of effects of GO on bacterial abundance and diversity is also in accordance with previous in vitro studies using a substrate for dairy animals and doses of GO ranging from 250 to 500 mg/L [13,14,15], suggesting that bacterial abundance is not adversely affected by this additive. Moreover, GO apparently modified the structure of bacterial populations [13,14,15], which is in contrast to the results of PCoA in this study, although this effect disappeared over time [15]. The lack of effects of GO on bacterial abundance and diversity could be seen as positive, since this microbial group is essential for rumen fermentation.

As already mentioned, the results of the present study suggest that the effects of GO on protozoal, fungi, and archaeal populations were less marked over time, which might indicate a possible adaptation of microorganisms to the additive. In contrast, other authors have described that the negative effects of GO on rumen protozoa were more marked over time in sheep after adding either 500 or 750 mg of GO/kg DM [16] or even that no effects were observed on protozoal populations in batch cultures using 250 mg/L of GO [14,15]. In partial agreement with our results, GO reduced archaea abundance in 24 h and 18-day fermentations in batch cultures [13,14,15], although these effects were also observed over time. Moreover, the lower relative abundance of *F. succinogenes* observed in this study at the end of P2 has also been reported previously in vitro, after adding doses of GO ranging from 250 to 500 mg/L in batch cultures [13,14,15]. As mentioned in the introduction, the contrasting results observed among studies might be related to factors such as differences in the type of experiment (in vitro or in vivo), the fermentation system, basal diet, GO composition and dose, or length of supplementation, among others. Even so, the results of the present study highlight the ability of the rumen ecosystem to adapt to different conditions within the rumen.

It is noteworthy that the reduction caused by GO on methane production in this study was more marked in P1 (28% reduction) than in P2 (11% reduction), which could be partly explained by the apparent adaptive response of archaea and protozoa to the additive over time. Rumen methanogens are involved in an ecto- and endosymbiotic relationship with anaerobic protozoa, and this association is considered one of the most active communities in the rumen methanogenesis [45,46]. Concerning this symbiotic relationship, the reduction in the DNA abundance of protozoa in SOL and LIQ phases at the end of P1, as well as in the relative abundance of archaea in LIQ, could lead to a greater reduction in methane production observed in P1 compared to P2. These results, along with the maintained changes in VFA profile caused by GO, suggest that 180 mg/L of this additive might reduce methane production not only by affecting archaea and protozoa populations but also by redirecting metabolic H_2_ to other fermentation pathways, such as propionate production, which is a hydrogen sink in the rumen [47]. Reducing enteric methane emissions without affecting ruminants’ productive performance is desirable to reduce their global warming effects and enhance feed conversion efficiency [1].

### 4.2. Cinnamaldehyde

Supplementation with CIN only increased the methane/total VFA ratio in P1, but in P2, CIN-fermenters showed increased ADF disappearance and acetate/propionate ratios compared with unsupplemented fermenters. Moreover, protozoal DNA concentration in the SOL phase decreased after CIN addition at the end of P1, whereas at the end of P2, CIN reduced bacterial and protozoal DNA concentrations in SOL and LIQ phases, respectively. These results indicate that the effects of CIN on rumen fermentation and microbial populations appear to be slightly more marked over time, which contrasts with the increasing effect of CIN in bacterial diversity in the LIQ phase at the end of P1 but not at the end of P2. Additionally, the increase caused by CIN in methane/total VFA and acetate/propionate ratios in P1 and P2, respectively, suggest that fermentation is less efficient when CIN is added into the fermenters. Notwithstanding, one of the most important findings of this study is the greater microbial protein synthesis and its efficiency observed in CIN-supplemented fermenters compared to CON ones. Concerning the results of CIN on MPS, it is difficult to explain why bacterial DNA concentration in SOL decreased at the end of P2 after supplementation with CIN, whereas MPS increased in the SOL phase at the same time. These controversial results could be related to the method used to quantify MPS (using ^15^N as a microbial marker) and bacterial DNA (qPCR), as these methods show differences in the way the microorganisms were recovered from the samples.

The absence of effects of CIN on diet disappearance and VFA production were in agreement with previous results observed in continuous cultures fed a mixed diet for dairy ruminants after 7 to 10 days on treatment [10,18], although reductions in organic matter and NDF digestibility were observed by adding CIN at 500 mg/L [48]. Some studies also reported that CIN modified VFA molar proportions by reducing either acetate proportion or the acetate/propionate ratio [10,18]. In contrast, Mateos et al. [11] observed that 180 mg/L of CIN increased the acetate/propionate ratio in batch cultures, which agrees with our results. Nevertheless, the controversial results observed among studies might be due to different factors such as the fermentation system, the dose of CIN, or the basal diet used, as already discussed.

Cinnamaldehyde did not significantly affect NH_3_-N production in the present study. Some previous in vitro studies also reported no effects of CIN on NH_3_-N concentration [10,48], although others observed a reduction [11,18]. Concerning MPS, Busquet et al. [10] observed no effects of 300 mg of CIN/L on MPS and its efficiency in continuous cultures, whereas Tager and Krause [48] reported a reduction, which was probably related to the high dose used by these authors (500 mg/L). Contrastingly, Cantet et al. [19] reported that 125 mg of CIN/d in dairy cows reduced milk urea nitrogen and increased the conversion efficiency of dietary nitrogen into milk protein nitrogen, which agrees with the higher MPS observed in this study. Moreover, Chapman et al. [17] observed a reduction in urinary urea N and total purine derivatives after supplementing the diet of dairy cows with 2 mg/kg of BW, without another effect on milk performance and rumen fermentation parameters. Although the effect of CIN on nitrogen metabolism in the rumen is difficult to explain, Sahan [49] studied the influence of cinnamon essential oil on in vitro protozoal activity, determined by the potentiality of rumen protozoa to digest 14C-labeled bacteria, and he observed a lower degradation of bacterial by protozoa after adding a dose of 5000 mg/kg of cinnamon essential oil. The results of this study suggest that CIN supplementation may increase the supply of microbial protein to the host animal, which is a positive and very interesting result. Since microbial protein represents the main source of amino acids for ruminants, improving MPS and its efficiency is one of the main goals in ruminant nutrition.

As mentioned before, the effects of CIN on bacterial diversity and microbial populations appeared to be less marked from P1 to P2 in this study. Similarly, results of PCoA showed differences in the structure of bacterial populations between CON and CIN-fermenters at the end of P1 but not at the end of P2. There is only limited information about the potential effects of CIN on rumen microbial populations. The abundance of total bacteria in batch cultures, as well as that of protozoa and *F. succinogenes* in the rumen of steers, were adversely affected by the use of cinnamon EO containing between 57% and 70% of CIN [50,51]. Although these results partially agree with those of the present study, Khorrami et al. [49] also reported that supplementing the diet of steers with CIN (5.7 g of cinnamon essential oil/d) decreased the relative abundance of archaea in the rumen. As cinnamon essential oil contains other compounds besides CIN, their combination might have a synergistic effect on certain microbial populations. However, the results of this study are only interpreted as the use of pure CIN and not cinnamon essential oil.

## 5. Conclusions

In conclusion, the results of the present study indicate that 180 mg/L of GO or CIN can positively modify rumen fermentation patterns and microbial protein synthesis, respectively, in Rusitec fermenters fed a diet formulated for dairy animals. Results of GO suggest an adaptive response of protozoa, fungi, and archaea to the additive, but this compound reduced methane production and promoted a more efficient rumen fermentation, maintaining these effects over time. Moreover, CIN increased MPS after 15 days of incubation, which suggests a greater protein supply to the animal. Nevertheless, more research is necessary in order to verify if these results are replicable in vivo, as well as to determine whether it is possible to minimize the consequences of the adaptive response of rumen microbial populations to GO in order to maintain a significant reduction in methane production during long time periods.

## Figures and Tables

**Table 1:** Ingredients and chemical composition of the diet for donor sheep and the diet incubated in the Rusitec fermenters.

Item	g/kg DM ^1^
**Ingredients**	
Alfalfa hay	500
Barley	199
Maize Soybean meal	96.0
	71.0
Lupins	60.0
Oat	31.5
Full-fat soybean	15.0
Calcium carbonate (CaCO_3_)	6.9
Sugarcane molasses	5.0
NaCl	3.5
Dicalcium phosphate (CaHPO_4_)	2.1
Mineral/vitamin premix ^2^	10.0
**Chemical composition**	
Organic matter	935
Crude protein	176
Neutral detergent fibre ^3^	368
Acid detergent fibre ^3^	162
Ether extract	26
Non-structural carbohydrates	355

^1^ DM: dry matter ^2^ Declared composition (g/kg mineral/vitamin premix): Vitamin A, 600,000 IU; Vitamin D3, 120,000 IU; Vitamin E, 1 g; Vitamin B1, 33 mg; Niacine, 1.5 g; S, 5 g; IK, 300 mg; SO_4_Fe, 1 g; ZnO, 4 g; MnO, 2 g; CoSO_4_, 60 mg; Na_2_SeO_3_, 30 mg; Ethoxyquin, 30 mg. ^3^ Expressed exclusive residual ash.

**Table 2:** Effects of adding 180 mg/L of either garlic oil or cinnamaldehyde in P1 (7 to 9 days of incubation) on diet disappearance, in vitro rumen fermentation parameters, and enzymatic activity in Rusitec fermenters fed a 50:50 forage/concentrate diet (*n* = 4).

Item	Treatment			SEM ^4^	*p*-Value
	CON ^1^	GO ^2^	CIN ^3^		
Diet disappearance (g/kg DM ^5^)					
Dry matter	640	622	640	10.9	0.08
Organic matter	633	614	632	11.2	0.09
Neutral detergent fibre	457	439	465	16.2	0.17
Acid detergent fibre	302	249 *	302	16.9	<0.001
pH	6.40	6.51	6.45	0.078	0.24
NH_3_-N ^6^ (mg/day)	234	221	215	9.9	0.07
Total VFA ^7^ (mmol/day)	113	106	109	3.8	0.10
Molar proportions (mol/100 mol)					
Acetate	55.2	50.8 *	55.7	0.84	<0.001
Propionate	16.0	19.1 *	15.3	0.61	<0.001
Butyrate	16.4	15.0 *	16.2	0.38	0.002
Isobutyrate	1.19	1.32	1.16	0.090	0.08
Valerate	4.90	6.61	4.61	0.315	<0.001
Isovalerate	3.27	2.37 *	3.67	0.271	<0.001
Caproate	3.06	4.80 *	3.40	0.261	<0.001
Acetate/propionate	3.46	2.70 *	3.64	0.111	<0.001
Methane (mmol/day)	23.1	16.6 *	26.3	1.59	<0.001
Methane/VFA (mol/mol)	0.20	0.17 *	0.24 *	0.017	<0.001
Enzymatic activity ^8^					
Amylase	285	305	315	30.3	0.55
Xylanase	696	673	642	42.4	0.67
Endoglucanase	84.0	98.9	96.9	8.76	0.49

^1^ CON: control; ^2^ GO: garlic oil; ^3^ CIN: cinnamaldehyde; ^4^ SEM: standard error of the mean; ^5^ DM: dry matter; ^6^ NH_3_-N: ammonia nitrogen; ^7^ VFA: volatile fatty acids; ^8^ Amylase and endoglucanase activities are expressed as nanomol of glucose released by 1 mL of liquid fermenters’ content in 1 min at 39 °C and pH 6.5 from soluble starch or carboxymethylcellulose, respectively. Xylanase activity is expressed as nanomol of xylose liberated from oat spelt xylan by 1 mL of liquid fermenters’ content in 1 min at 39 °C and pH 6.5. * Indicates significant differences between additive and control (*p* < 0.05).

**Table 3:** Effects of adding 180 mg/L of either garlic oil or cinnamaldehyde in P2 (12 to 14 days of incubation) on diet disappearance, in vitro rumen fermentation parameters, and enzymatic activity in Rusitec fermenters fed a 50:50 forage/concentrate diet (*n* = 4).

Item	Treatment			SEM ^4^	*p*-Value
	CON ^1^	GO ^2^	CIN ^3^		
Diet disappearance (g/kg DM ^5^)					
Dry matter	619	623	628	13.1	0.73
Organic matter	615	617	621	13.1	0.82
Neutral detergent fibre	459	446	473	16.7	0.15
Acid detergent fibre	277	274	327 *	22.5	0.01
pH	6.51	6.51	6.49	0.082	0.93
NH_3_-N ^6^ (mg/day)	210	221	195	9.8	0.01
Total VFA ^7^ (mmol/day)	103	100	98	3.7	0.31
Molar proportions (mol/100 mol)					
Acetate	54.5	53.6 *	53.7	0.45	0.01
Propionate	14.9	17.1 *	13.4 *	0.37	<0.001
Butyrate	17.2	14.6 *	17.6	0.48	<0.001
Isobutyrate	1.22	1.32 *	1.23	0.035	<0.01
Valerate	4.85	5.86 *	4.82	0.170	<0.001
Isovalerate	4.30	2.62 *	5.99 *	0.418	<0.001
Caproate	3.06	4.90 *	3.30	0.271	<0.001
Acetate/propionate	3.67	3.14 *	4.01 *	0.096	<0.001
Methane (mmol/day)	27.5	24.5 *	27.4	0.98	<0.001
Methane/VFA (mol/mol)	0.27	0.24 *	0.28	0.007	<0.001
Enzymatic activity ^8^					
Amylase	259	242	232	25.5	0.75
Xylanase	491	511	443	25.0	0.17
Endoglucanase	67.6	63.7	61.9	3.17	0.22

^1^ CON: control; ^2^ GO: garlic oil; ^3^ CIN: cinnamaldehyde; ^4^ SEM: standard error of the mean; ^5^ DM: dry matter; ^6^ NH_3_-N: ammonia nitrogen; ^7^ VFA: volatile fatty acids; ^8^ Amylase and endoglucanase activities are expressed as nanomol of glucose released by 1 mL of liquid fermenters’ content in 1 min at 39 °C and pH 6.5 from soluble starch or carboxymethylcellulose, respectively. Xylanase activity is expressed as nanomol of xylose liberated from oat spelt xylan by 1 mL of liquid fermenters’ content in 1 min at 39 °C and pH 6.5. * Indicates significant differences between additive and control (*p* < 0.05).

**Table 4:** Effects of adding 180 mg/L of either garlic oil or cinnamaldehyde on microbial protein synthesis (MPS) in solid content of nylon bags and effluents, total MPS, and the efficiency of MPS in Rusitec fermenters fed a 50:50 forage/concentrate diet (*n* = 4).

Item	Treatment			SEM ^4^	*p*-Value
	CON ^1^	GO ^2^	CIN ^3^		
Microbial protein synthesis (mg N/day)					
Solid	128	137	160 *	6.0	0.02
Liquid	110	110	128	5.0	0.05
Total	238	247	288 *	5.6	<0.001
Efficiency of microbial growth ^5^	27.5	30.5	35.5 *	0.99	<0.01

^1^ CON: control; ^2^ GO: garlic oil; ^3^ CIN: cinnamaldehyde; ^4^ SEM: standard error of the mean; ^5^ Expressed as mg microbial N per g OM apparently fermented; OM apparently fermented was calculated from VFA production according to Demeyer [42]. * Indicates significant differences between additive and control (*p* < 0.05).

**Table 5:** Effects of adding 180 mg/L of either garlic oil or cinnamaldehyde at the end of P1 (day 10) on the number of peaks, Shannon’s diversity index, the concentration of bacterial and protozoal DNA, and the relative abundance of *F. succinogenes*, *R. flavefaciens*, *R. albus*, fungi, and archaea in solid and liquid phases of Rusitec fermenters fed a 50:50 forage/concentrate diet (*n* = 4).

Phase	Item	Treatment			SEM ^4^	*p*-Value
		CON ^1^	GO ^2^	CIN ^3^		
Solid	Number of peaks	35.5	37.8	39.0	2.00	0.91
	Shannon index	3.57	3.63	3.64	0.053	0.98
	Total bacteria (μg DNA/g DM ^5^)	1900	1349	3138	911.8	0.41
	Total protozoa (μg DNA/g DM ^5^)	28.95	13.65 *	7.55 *	3.333	<0.01
	*Fibrobacter succinogenes* ^6^	10.41	11.31	9.50	3.888	0.95
	*Ruminococcus flavefaciens* ^6^	0.14	0.14	0.19	0.041	0.67
	*Ruminococcus albus* ^6^	10.29	18.64	13.72	4.520	0.46
	Fungi ^6^	3.09	0.675 *	1.94	0.442	0.02
	Archaea ^6^	0.37	0.20	0.52	0.090	0.10
Liquid	Number of peaks	41.5	36.0	51.3 *	1.75	0.001
	Shannon index	3.72	3.58	3.93 *	0.043	0.002
	Total bacteria (μg DNA/mL)	115	1160.02	136	10.0	0.33
	Total protozoa (μg DNA/mL)	30.47	3.16 *	13.93	5.390	0.03
	Fungi ^6^	0.88	0.02	0.25	0.217	0.06
	Archaea ^6^	0.24	0.07 *	0.24	0.034	0.01

^1^ CON: control; ^2^ GO: garlic oil; ^3^ CIN: cinnamaldehyde; ^4^ SEM: standard error of the mean; ^5^ DM: dry matter; ^6^ Expressed as relative abundance to the absolute quantification of total bacteria DNA concentration as 2^−(CT target − CT total bacteria)^. * Indicates significant differences between additive and control (*p* < 0.05).

**Table 6:** Effects of adding 180 mg/L of either garlic oil or cinnamaldehyde at the end of P2 (day 15) on the number of peaks, Shannon’s diversity index, the concentration of bacterial and protozoal DNA, and the relative abundance of *F. succinogenes*, *R. flavefaciens*, *R. albus*, fungi, and archaea in solid and liquid phases of Rusitec fermenters fed a 50:50 forage/concentrate diet (*n* = 4).

Phase	Item	Treatment			SEM ^4^	*p*-Value
		CON ^1^	GO ^2^	CIN ^3^		
Solid	Number of peaks	37.8	35.5	41.3	1.77	0.14
	Shannon index	3.63	3.57	3.71	0.045	0.14
	Total bacteria (μg DNA/g DM ^5^)	2243	1564	1111 *	258.0	0.047
	Total protozoa (μg DNA/g DM ^5^)	27.54	0.93	2.82	8.069	0.09
	*Fibrobacter succinogenes* ^6^	4.60	0.44 *	2.68	0.527	0.01
	*Ruminococcus flavefaciens* ^6^	0.17	0.23	0.17	0.032	0.37
	*Ruminococcus albus* ^6^	14.37	21.89	8.28	2.188	0.01
	Fungi ^6^	5.45	0.03	29.77	10.54	0.18
	Archaea ^6^	0.32	0.10	0.65	0.103	0.02
Liquid	Number of peaks	41.0	45.5	51.5	3.05	0.21
	Shannon index	3.69	3.81	3.94	0.074	0.20
	Total bacteria (μg DNA/mL)	101	138	85	13.5	0.07
	Total protozoa (μg DNA/mL)	18.82	1.04	3.53 *	3.645	0.049
	Fungi ^6^	0.31	0.00	0.07	0.112	0.19
	Archaea ^6^	0.17	0.08	0.25	0.040	0.06

^1^ CON: control; ^2^ GO: garlic oil; ^3^ CIN: cinnamaldehyde; ^4^ SEM: standard error of the mean; ^5^ DM: dry matter; ^6^ Expressed as relative abundance to the absolute quantification of total bacteria DNA concentration as 2^−(CT target − CT total bacteria)^. * Indicates significant differences between additive and control (*p* < 0.05).

## Data Availability

The datasets used and/or analyzed during the current study are available from the corresponding author upon reasonable request.
